# Bridging the Gap: Improving Acute Ischemic Stroke Outcomes with Intravenous Thrombolysis Prior to Mechanical Thrombectomy

**DOI:** 10.3390/neurolint16060090

**Published:** 2024-10-22

**Authors:** Jessica Seetge, Balázs Cséke, Zsófia Nozomi Karádi, Edit Bosnyák, László Szapáry

**Affiliations:** 1 Stroke Unit, Department of Neurology, University of Pécs, 7624 Pécs, Hungary; j.seetge@gmx.de (J.S.); karadi.zsofia@pte.hu (Z.N.K.); bosnyak.edit@pte.hu (E.B.); 2 Department of Emergency Medicine, University of Pécs, 7624 Pécs, Hungary; cseke.balazs@pte.hu

**Keywords:** acute ischemic stroke, intravenous thrombolysis, mechanical thrombectomy, bridging

## Abstract

Background/Objectives: Current guidelines recommend intravenous thrombolysis (IVT) followed by mechanical thrombectomy (MT) for patients with acute ischemic stroke (AIS) caused by large vessel occlusion (LVO). This combined approach, known as bridging therapy (BT), is believed to increase the likelihood of a favorable functional outcome when administered within 4.5 h of symptom onset. However, the benefits of BT over direct mechanical thrombectomy (d-MT) remain debated. This study aimed to compare the outcomes of AIS-LVO patients undergoing MT within 6 h of symptom onset, with and without prior IVT. Methods: Within the prospective Transzlációs Idegtudományi Nemzeti Laboratórium (TINL) STROKE-registry, AIS-LVO patients admitted to the Department of Neurology, University of Pécs between February 2023 and June 2024 were investigated. The primary endpoint was the proportion of patients reaching functional independence at 90 days, defined as a modified Rankin Scale (mRS) score of 0–2. Secondary endpoints included clinical improvement at 72 h (National Institute of Health Stroke Scale [NIHSS] score of ≤1 or a change from baseline [ΔNIHSS] of ≥4) and successful recanalization (modified Thrombolysis in Cerebral Infarction [mTICI] score ≥ 2). Safety outcomes were evaluated based on thrombus migration and intracranial hemorrhage (ICH). Results were compared using linear and logistic regression analyses adjusted for baseline variables. Results: Of 82 patients, 51 (62.2%) received BT, while 31 (37.8%) underwent d-MT. The BT group showed a significantly higher rate of functional independence (45.7% vs. 17.2%, *p* = 0.014) and a lower 90-day mortality rate (13.7% vs. 35.5%, *p* = 0.029). Multivariate analysis revealed that IVT was independently associated with favorable functional outcomes (*p* = 0.011) and reduced mortality (*p* = 0.021). No significant differences were observed in terms of clinical improvement at 72 h, successful recanalization, thrombus migration, or hemorrhagic transformation between the groups. Conclusions: This study supports current guidelines recommending BT for thrombectomy-eligible AIS-LVO patients, offering new insights into the ongoing clinical debate.

## 1. Introduction

Acute ischemic stroke (AIS) caused by large-vessel occlusion (LVO) is a significant cause of morbidity and mortality worldwide [1]. Timely and effective intervention is critical to minimizing brain damage and improving patient outcomes [2]. LVOs, characterized by blockages in the proximal intracranial vessels, account for approximately 24% to 46% of all acute ischemic strokes [3]. While ischemic changes can occur within minutes, the extent of infarcted tissue is primarily influenced by the severity and duration of hypoperfusion, with collateral circulation playing a crucial role in stroke progression [4].

Due to advancements in neuroimaging and interventional devices, mechanical thrombectomy (MT) has become the standard treatment for AIS-LVO, allowing for the removal of the obstructing clot from the affected artery. Current guidelines advocate for intravenous thrombolysis (IVT) with recombinant tissue plasminogen activator (rtPA) within 4.5 h of symptom onset as the first-line therapy for eligible patients, significantly increasing the likelihood of a favorable outcome [5].

Despite its efficacy, the use of IVT prior to MT (bridging therapy, BT) in clinical practice raises concerns. BT may delay the initiation of MT and introduce additional risks, such as distal embolization and hemorrhagic transformation, potentially compromising reperfusion rates and complicating the procedure. Each hour of delay in starting MT reduces the chance of achieving functional independence by 5.3%, raising the question of whether omitting IVT in cases of AIS-LVO could optimize treatment workflows and improve clinical outcomes [6]. Furthermore, individual patient characteristics are crucial in guiding treatment decisions, and tailoring therapies based on these variables may predict outcomes and mitigate unnecessary risks associated with IVT.

Several randomized controlled trials (RCTs) have provided conflicting evidence regarding the non-inferiority of direct mechanical thrombectomy (d-MT) compared to BT. However, these studies have exclusively focused on patients treated at ‘mothership’ hospitals, where all interventions are provided at a single comprehensive stroke center (CSC). To date, no RCTs have investigated the efficacy and safety of BT within the ‘drip-and-ship’ model, where IVT is administered at a primary stroke center (PSC) before transferring the patient to a thrombectomy-capable center, leaving uncertainty about the efficacy and safety of IVT in this context. Current guidelines recommend IVT before MT for eligible patients who are directly admitted to CSCs within 4.5 h of symptom onset [5]. However, there is limited evidence supporting the use of IVT in AIS-LVO patients arriving at PSCs without endovascular facilities, highlighting the need for further research to determine the optimal treatment approach for these patients.

In this study, we aimed to evaluate the efficacy and safety of IVT before MT compared to d-MT within 6 h of symptom onset in patients with AIS-LVO who presented to our PSC, thereby contributing to the ongoing clinical debate.

## 2. Materials and Methods

### 2.1. Study Design and Patient Population

We conducted a retrospective study using data from our prospective Transzlációs Idegtudományi Nemzeti Laboratórium (TINL) STROKE-registry. This registry includes comprehensive demographic and clinical data such as age, sex, chronic comorbidities, cardiovascular risk factors, previous medications, premorbid functional status, imaging parameters, stroke characteristics, and functional and procedural outcomes.

From February 2023 to June 2024, 951 consecutive adult patients were admitted to the Department of Neurology, University of Pécs. A total of 856 patients were excluded for the following reasons: acute hemorrhagic stroke (AHS) (*n* = 59), transient ischemic attack (TIA) (*n* = 122), treatment with only IVT or supportive care (SC) (*n* = 557), onset-to-puncture time exceeding 6 h (*n* = 111), and absolute contraindications for IVT (*n* = 13). Ultimately, 82 patients were included in the study and were divided into the BT group (*n* = 51) and the d-MT group (*n* = 31).

**Figure 1:**
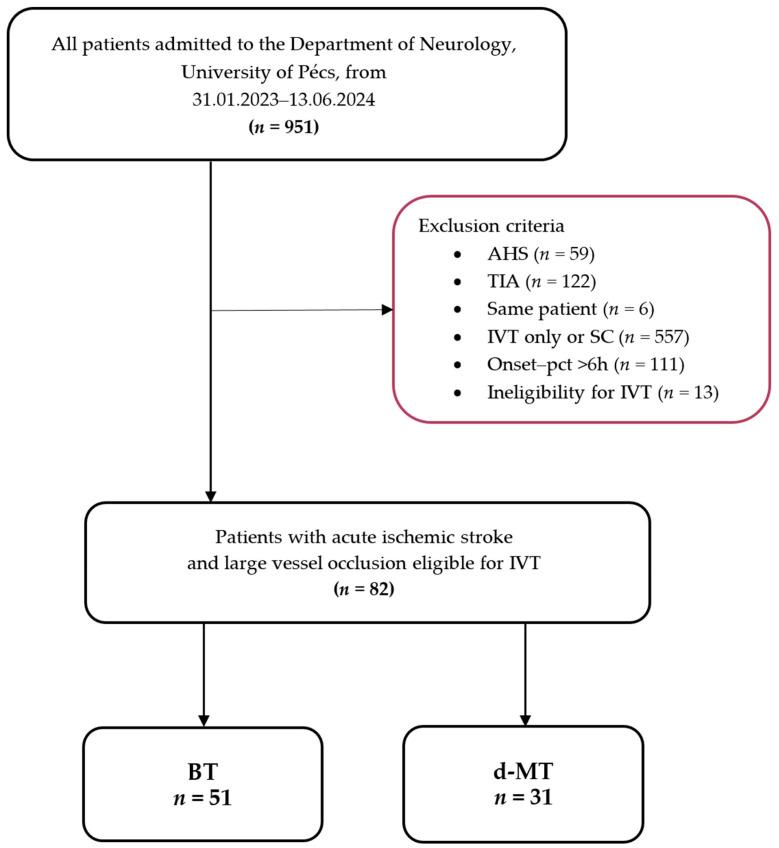
Flowchart of patients included in the study. Abbreviations: AHS = acute hemorrhagic stroke, TIA = transient ischemic attack, IVT = intravenous thrombolysis, SC = standard care, onset-pct = onset-puncture time, BT = bridging therapy, d-MT = direct mechanical thrombectomy.

The inclusion criteria were (1) acute ischemic stroke caused by large vessel occlusion, (2) triaged at the primary stroke center using multimodal CT imaging, including non-contrast computed tomography (NCCT), CT angiography (CTA), or CT perfusion (CTP), immediately after clinical evaluation, (3) occlusion located in the intracranial internal carotid artery (ICA), middle cerebral artery (M1, M2, and proximal M3 segments), the A1 segment of the anterior cerebral artery (ACA), or the P1 segment of the posterior cerebral artery (PCA), and (4) subsequent mechanical thrombectomy within 6 h of symptom onset. The flow chart is shown in Figure 1.

### 2.2. Data Collection and Measurements

Brain and vessel imaging findings included early ischemic signs assessed using the manual Alberta Stroke Program Early Computed Tomography Score (mASPECTS), the occlusion site, and the graded multiphase computed tomography angiography (mCTA) collateral score, evaluated by a radiologist or neuroradiologist. The stroke mechanism was classified according to the Trial of Org 10172 in Acute Stroke Treatment (TOAST) criteria. Treatment times were recorded for the intervals from onset to admission, door-to-needle, and door-to-puncture. Stroke severity was assessed by a stroke neurologist using the National Institute of Health Stroke Scale (NIHSS) score at admission and 72 h post-treatment.

Final treatment decisions were made case-by-case at the discretion of the neurologist and neurointerventionalist on duty. The primary reasons for not administering IVT to eligible patients included exclusion criteria from previous guidelines, which are no longer present in current guidelines, such as a time window exceeding 4.5 h (*n* = 2), or relative contraindications like early CT signs of ischemia (*n* = 7), known malignancy (*n* = 6), or being near the 4.5 h time window (*n* = 4). All contraindications are detailed in Table 1.

Patients in the BT group received rtPA at a dose of 0.9 mg/kg of body weight within 4.5 h of symptom onset, in line with national and international stroke guidelines. Standard laboratory and clinical inclusion and exclusion criteria for IVT were applied. IVT was initiated in the CT suite immediately after confirming the absence of bleeding. MT was performed following digital subtraction angiography (DSA) via a femoral artery approach, under either general anesthesia or conscious sedation, by board-certified interventional neuroradiologists. The choice of thrombectomy device, including aspiration techniques and stent retrievers, was left to the operator’s discretion.

Recanalization success was assessed by a neurointerventionalist using the modified Thrombolysis in Cerebral Infarction (mTICI) scale, categorized as 2a (partial filling < 50%), 2b (partial filling ≥ 50%) and 3 (complete perfusion). Repeated CT imaging was performed twenty-four hours post-treatment or in the event of deterioration to detect hemorrhagic transformation (HT). The type of intracerebral hemorrhage (ICH) was classified during hospitalization according to the European Cooperative Acute Stroke Study (ECASS) classification as hemorrhagic infarction (HI) and parenchymal hemorrhage (PH) or subarachnoid hemorrhage (SAH). Functional outcomes were evaluated using the modified Rankin Scale (mRS) through telephone interviews conducted 90 days after the intervention.

### 2.3. Outcome Parameters

The primary endpoint of this study was functional independence at 90 days, defined as a mRS score of 0–2, assessed by a physician or a trained and certified neurology nurse. Secondary endpoints included clinical improvement at 72 h, defined as a NIHSS score of ≤1 or an improvement from baseline (ΔNIHSS) of ≥4, and successful recanalization, defined as a mTICI score of ≥2b. Safety outcomes were evaluated based on thrombus migration and ICH. Missing NIHSS scores were retrospectively scored, and an mRS score of 0–5 at 30 days was considered missing.

### 2.4. Statistical Analyses

Data were analyzed using the Statistical Product and Service Solutions (SPSS) program (version 23). Independent continuous variables were assessed for normality using both descriptive and analytical criteria. Baseline characteristics were summarized using descriptive statistics. Continuous variables were described as mean ± standard deviation (SD) or median (with interquartile range [IQR]) values and compared using the Mann–Whitney U test. Categorical variables were displayed as numbers, frequencies, or percentages and compared using Fisher’s exact test.

Differences between groups in demographic, clinical, imaging, and procedural characteristics were evaluated using Fisher’s exact test for binary data. Multivariate linear and logistic regression analysis were conducted to evaluate predictors of functional and procedural outcomes, adjusting for baseline variables such as age, pre-mRS score, admission NIHSS score, mASPECTS, and mCTA collateral score. Additionally, logistic regression was performed to quantify the association of BT with mRS score when treated as a continuous variable to capture more subtle associations.

In light of the small sample size, bootstrapping analysis with 1000 resamples was used to validate the robustness of the findings.

All statistical tests were two-tailed, and odds ratios (OR) with 95% confidence intervals (CI) were reported. A *p*-value of <0.05 was considered statistically significant.

## 3. Results

### 3.1. Demographic and Clinical Characteristics

Eighty-two consecutive patients with AIS due to LVO were retrospectively reviewed. Among these, 51 patients (62.2%) received BT (47.1% male, median age 67 years [IQR, 33–89]) and 31 patients (37.8%) underwent d-MT who would have qualified for BT (35.5% male, median age 72 years [IQR, 44–93]). Clinical characteristics included a pre-mRS score of 0 (IQR, 0–5) for the BT group and 0 (IQR, 0–4) for the d-MT group, and an NIHSS score of 10 (IQR, 1–36) for the BT group and 9 (IQR, 0–39) for the d-MT group. Detailed demographic and clinical characteristics are presented in Table 2.

### 3.2. Imaging and Stroke Characteristics

There was no significant difference in imaging characteristics between the groups, except for a higher median mASPECTS in the BT group (9 [IQR, 5–10]) compared to the d-MT group (8 [IQR, 5–10], *p* = 0.027). Multivariate linear regression analysis revealed mCTA collateral score (*p* = 0.032), plasma sodium level (*p* = 0.008), and C-reactive protein (CRP) (*p* = 0.028) as significant predictors of mASPECTS. Collateral scores showed only numerical differences; excellent: 4–5 (72.0% vs. 65.4%), good: 2–3 (26.0% vs. 34.6%), and poor: 0–1 (2.0% vs. 0.0%). Significant predictors of an excellent mCTA collateral score included mASPECTS (*p* = 0.032), admission NIHSS score (*p* = 0.035), plasma glucose (*p* = 0.034), and sodium level (*p* = 0.023). The median door-to-needle time for the BT group was 43 min (IQR, 25–195), while the median door-to-puncture time was 126 min (IQR, 69–290) for BT and 112 min (IQR, 30–293) for d-MT. Detailed imaging and stroke characteristics are summarized in Table 3.

### 3.3. Functional and Procedural Outcomes and Predictors

**Figure 2:**
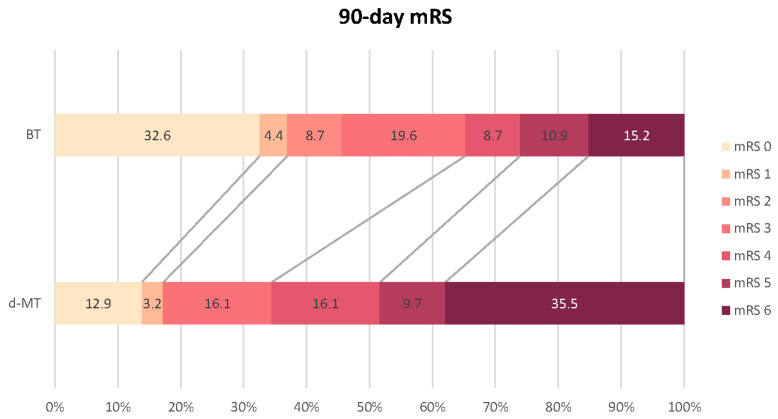
Distribution of 90-day mRS scores in the BT and d-MT groups. Abbreviations: mRS = modified Rankin Scale, BT = bridging therapy, d-MT = direct mechanical thrombectomy. Number values shown in both groups are rounded estimations. Number values for mRS 6 of both treatment groups are shown in white only to improve visibility and hold no difference from the other mRS percentage values.

At the 90-day follow-up, a significantly higher proportion of patients in the BT group (*n* = 46) achieved functional independence (mRS 0–2) compared to the d-MT group (*n* = 29) (45.7%; 95% CI: 31.03–60.34% vs. 17.2%; 95% CI: 5.17–34.48%, *p* = 0.014). Multivariate linear regression analysis identified BT (*p* = 0.011), pre-mRS score (*p* = 0.029), baseline NIHSS score (*p* = 0.015), 72 h NIHSS score (*p* < 0.001), age (*p* = 0.001), mASPECTS (*p* = 0.042), and history of dyslipidemia (*p* = 0.001) as significant predictors of favorable functional outcome. The 90-day mortality rate was significantly lower in the BT group (13.7%; 95% CI: 5.88–23.53% vs. 35.5%; 95% CI: 19.35–51.61%, *p* = 0.029). Protective factors against death within the first three months included BT (*p* = 0.021), higher mASPECTS (*p* = 0.002), and better collateral status (*p* = 0.010). In contrast, a higher pre-mRS score (*p* = 0.028), older age (*p* = 0.004), higher NIHSS score at admission (*p* = 0.003) and after 72 h (*p* < 0.001), and hyperglycemia (*p* = 0.032) were associated with increased mortality. Logistic regression analysis indicated that preceding IVT was independently associated with a favorable functional outcome and lower mortality at 90 days, reducing the odds of mRS scores > 2 by 74% (*p* = 0.019) and death by 70.4% (*p* = 0.028) compared to d-MT. Figure 2 illustrates the distribution of 90-day mRS scores in both treatment groups.

The BT group showed a trend towards lower NIHSS scores at 72 h, with a median score of 3 compared to 9 in the d-MT group (*p* = 0.095). However, statistical significance was not reached, likely due to the small sample size. Multiple linear regression analysis revealed that better neurological outcomes at 72 h were significantly associated with admission NIHSS score (*p* < 0.001), mASPECTS (*p* < 0.001), collateral status (*p* = 0.014), number of passes during MT (*p* = 0.019), history of dyslipidemia (*p* = 0.001), and plasma sodium levels (*p* = 0.047). Additionally, there was a trend towards better outcomes with preceding IVT (*p* = 0.090) and successful recanalization (*p* = 0.080).

There was no significant difference in clinical improvement at 72 h between the BT and d-MT groups (NIHSS score of 0 or 1: 33.3% vs. 16.1%, *p* = 0.124; ΔNIHSS ≥ 4: 45.1% vs. 29.0%, *p* = 0.169).

The rates of successful recanalization, as determined by mTICI grading scales, were comparable between the BT and d-MT groups (86.3% vs. 93.6%, *p* = 0.472). There was a numerical difference in thrombus migration and the incidence of ICH between the two groups, with the BT group showing a higher frequency of embolization (17.7% vs. 9.7%, *p* = 0.521) but a lower rate of ICH (3.9% vs. 12.9%, *p* = 0.193) compared to the d-MT group. Notably, both ICH events in the BT group were asymptomatic (aICH), whereas two out of four ICHs in the d-BT group were symptomatic (sICH). Detailed functional and procedural outcomes and their predictors are summarized in Table 4 and Table 5.

## 4. Discussion

In this study, we evaluated the benefits and risks associated with administering IVT prior to MT. By excluding patients who were not eligible for IVT, our linear and logistic regression analyses confirmed a positive predictive association between IVT administration and favorable functional outcome and reduced mortality at 90 days, without an increased risk of thrombus migration or near-term hemorrhagic complications.

### 4.1. Current Knowledge on Bridging Therapy vs. Direct Mechanical Thrombectomy

The optimal treatment approach for patients with AIS due to LVO remains a subject of debate. Recent RCTs and meta-analyses have yielded mixed evidence regarding the efficacy of BT compared to d-MT.

Early trials, such as the DIRECT-MT and the DEVT trials in China, demonstrated the non-inferiority of d-MT compared to BT with alteplase in IVT-eligible patients [7,8]. However, these studies were limited by generous non-inferiority margins and extended door-to-IVT times [9]. Similarly, the SKIP trial conducted in Asia failed to show the non-inferiority of d-MT compared to BT [10].

The MR CLEAN-NO IV trial aimed to assess the superiority of d-MT over BT but found no significant difference in functional outcomes at 90 days, neither in terms of superiority or non-inferiority [11]. The SWIFT-DIRECT and DIRECT-SAFE trials evaluated the non-inferiority of d-MT for ICA and M1 occlusions [12,13]. The DIRECT-SAFE trial uniquely included M2 and basilar artery occlusions [13], making it the only one of the six RCTs to address posterior circulation LVOs.

A data analysis of over 2300 patients across the cited RCTs did not confirm the non-inferiority of MT alone compared to bridging therapy based on the predefined non-inferiority margin of 1.3% [14]. As a result, the latest guidelines recommend IVT preceding MT over d-MT for AIS patients with anterior LVO within 4.5 h of symptom onset who are directly admitted to a thrombectomy-capable center [14].

This recommendation contrasts with findings from two major meta-analyses of observational studies, which suggested that BT is associated with higher rates of successful recanalization, better functional outcomes, and lower 90-day mortality without an increased risk of sICH [15,16]. Nonetheless, these results should be interpreted cautiously, as many d-MT patients were ineligible for IVT, potentially placing them at higher risk for unfavorable outcomes and hemorrhagic complications. Observational data from two small single-center studies involving patients eligible for BT but treated with MT alone indicate that d-MT is equally effective when patients receive immediate treatment at a stroke center with rapid access to interventional procedures [17,18].

The six recent RCTs investigating the impact of IVT with alteplase before MT focused exclusively on ‘mothership’ patients with anterior circulation LVOs who were eligible for both treatments, with IVT administered within 4.5 h of stroke onset. These findings should not be generalized to patients receiving IVT at other centers (‘drip-and-ship’, ‘drip-and-drive’, or ‘drip-and-fly’) [9].

Systematic reviews and meta-analyses of observational data support the current guideline recommending BT for all IVT-eligible LVO ‘drip-and-ship’ patients [19,20]. Although the quality of evidence supporting the recommendation to withhold IVT in MT-eligible patients arriving at PSCs without thrombectomy facilities is low, no RCTs address this specific question, and such studies are unlikely to be conducted due to the lack of support from trials involving patients directly admitted to thrombectomy-capable centers [9]. However, direct access to MT is limited to a minority of LVO patients [21], and withholding IVT at PSCs may result in the denial of reperfusion therapy for some patients, especially those who reach CSCs outside the time window for endovascular therapies or those with unsuccessful MT [22,23].

### 4.2. Study Findings

A subgroup analysis of the SELECT cohort study revealed that patients with LVO treated with BT at PSCs before being transferred to MT-capable centers showed higher rates of excellent functional outcomes (mRS 0–1) compared to IVT-eligible patients receiving d-MT [24]. Our study produced similar results, showing significantly higher rates of good functional outcomes and lower 90-day mortality in the BT group compared to the d-MT group, contrasting with the findings of previous studies by Broeg-Morvay et al. and Weber et al. [17,18]. The sustained beneficial effect of IVT beyond the critical event of recanalization during thrombectomy may be attributed to the prolonged pharmacologic impact of rtPA on cerebral microcirculation. A randomized placebo-controlled clinical trial involving 121 LVO patients found that intra-arterial administration of rtPA after thrombectomy with successful reperfusion increased the likelihood of achieving an excellent functional outcome at 90 days [25].

Our study observed a trend toward lower median NIHSS scores in patients with BT at 72 h post-treatment. This contrasts with Broeg-Morvay et al., who reported a trend toward neurological improvement in NIHSS scores from baseline at 90 days in the d-MT group [17]. One possible explanation could be that early recovery trends may change when observed over a longer time period.

A 2021 multicenter retrospective cohort study found significantly higher rates of successful recanalization in IVT-eligible d-MT patients compared to BT patients (92.0% vs. 81.9%) [15]. Other studies have also shown a trend towards mTICI ≥ 2b in d-MT patients eligible for both treatments [26,27]. In contrast, Broeg-Morvay et al. reported no significant difference in the recanalization rates between the groups [17]. Similarly, our study demonstrated comparable rates of successful recanalization in both BT and d-MT patients, which may reflect consistent procedural standards and similar patient characteristics within our cohort.

Various studies have reported different incidences of ICH following BT and d-MT. Broeg-Morvay et al. reported higher aICH rates in the BT group [17], while Kurminas et al. and Kolahchi et al. observed significantly higher sICH incidences in BT patients [28,29], contrasting with Kass-Hout et al., who found no difference in hemorrhagic complications between the groups [30]. In our study, the d-MT group had numerically higher ICH rates, particularly sICH. This increased rate could be related to anticoagulation therapy, as d-MT patients were more likely to be anticoagulated on admission (16.1% vs. 7.8%, *p* = 0.288). However, patients in both groups were not under the effect of anticoagulants during MT, and there is no current evidence suggesting an association between anticoagulation and bleeding in d-MT patients.

### 4.3. Advantages and Disadvantages of Bridging Therapy

There are several considerations in favor of administering IVT before MT. Pretreatment with IVT can alter the composition of the clot, making it more susceptible to MT, potentially reducing the number of stent retriever passes required [31,32,33,34], and even achieving early reperfusion, which could negate the need for MT altogether [35]. Moreover, IVT may address distal microemboli that are difficult to reach with MT or residual occlusions after MT, benefiting patients where MT is delayed, not feasible, or insufficient to achieve complete reperfusion [36].

Conversely, the primary theoretical benefits of withholding IVT for LVO patients revolve around concerns about efficacy and safety. Evidence suggests that IVT is ineffective in the majority of LVO patients, with recanalization rates as low as 4–20% [37,38], and often does not result in significant symptom improvement [39]. Safety concerns include the risk of bleeding and embolization associated with IVT [35]. Additionally, logistic delays in transferring patients to a CSC and economic considerations must be taken into account [40,41].

### 4.4. Predictors for Administering IVT Before MT

Recent studies suggest that baseline variables could guide the decision to administer IVT before MT, with factors such as stroke severity, mASPECTS, and collateral status influencing the potential benefit [42]. For example, the limited efficacy of IVT in patients with higher NIHSS scores is likely due to the correlation between elevated NIHSS scores and major vessel occlusion, as IVT is three times more effective for M2 than ICA occlusions [43]. Contrasting findings, such as the ETIS study, reported higher functional independence at 90 days, better early neurologic improvement, and successful reperfusion in BT patients with large infarct cores (mASPECTS 0–5), suggesting even patients with substantial initial infarct burden might benefit from BT [44]. However, whether these characteristics reliably predict the harm or benefit of IVT in MT-eligible patients remains uncertain, underlining the need for ongoing research to refine treatment protocols and optimize outcomes for AIS-LVO patients.

### 4.5. Limitations

This study has several limitations, including a relatively small sample size from a single center and the non-randomized nature of the comparison, which introduces potential bias. Our observation of improved functional outcomes following BT was derived from a retrospective analysis of a prospective registry with partially imbalanced groups. Despite efforts to adjust for baseline differences, unmeasured confounding variables may still influence the outcomes. Treatment allocation was based on clinical judgment, leading to potential confounding by indication, which could skew the results toward a poorer prognosis. Expanding the study to include multiple centers could enhance the generalizability of our findings by capturing a broader range of patient characteristics and clinical practices, thereby minimizing institutional bias and offering a stronger foundation for future research.

## 5. Conclusions

In conclusion, our study findings indicate that BT is independently associated with improved functional outcomes and reduced 90-day mortality rates compared to d-MT. The lack of significant differences in recanalization success or safety measures between the groups implies that the benefits of BT are likely due to the additional therapeutic effects of IVT administered prior to MT. These results align with current guidelines recommending BT for IVT-eligible AIS-LVO patients undergoing MT within 6 h of symptom onset. Given the limitations of our study, these findings should be viewed as hypothesis-generating, and further research, particularly through RCTs, is required to determine whether d-MT could be as effective as BT in specific patient populations. Such research could refine treatment protocols and enhance decision-making in the management of acute ischemic stroke.

## Figures and Tables

**Table 1:** Absolute and relative contraindications for IVT in d-MT patients.

	d-MT (*n* = 44)
Absolute contraindications, *n* (%)	
Active anticoagulant use Intracranial hemorrhage	12 (27.3%) 1 (2.3%)
Relative contraindications, *n* (%)	
Non-axial Intracranial neoplasm Known malignancy Severe comorbidities Close time-window Early CT signs of ischemia Improving stroke symptoms Denied permission Unknown reason	2 (4.6%) 6 (13.6%) 1 (2.3%) 4 (9.1%) 7 (15.9%) 1 (2.3%) 1 (2.3%) 7 (15.9%)

IVT = intravenous thrombolysis, d-MT = direct mechanical thrombectomy; Time window > 4.5 h (*n* = 2).

**Table 2:** Demographic and clinical characteristics of the 31 patients in the d-MT group without contraindications for IVT and 51 patients treated with BT.

	BT (*n* = 51)	d-MT (*n* = 31)	*p*-Value/OR [95% CI]
Demographic characteristics			
Age, years, median (IQR)	67 (33–89)	72 (44–93)	*p* = 0.250
Sex, male, *n* (%)	24 (47.1%)	11 (35.5%)	OR = 1.62 [0.65–4.05], *p* = 0.361
Medical history, *n* (%)			
Current smoking Alcohol Hypertension Diabetes mellitus Atrial fibrillation * Dyslipidemia	18 (35.3%) 19 (37.3%) 41 (80.4%) 9 (17.7%) 16 (31.4%) 40 (78.4%)	12 (38.7%) 9 (29.0%) 27 (87.1%) 7 (22.6%) 7 (22.6%) 21 (67.7%)	OR = 0.86 [0.34–2.17], *p* = 0.815 OR = 1.45 [0.56–3.79], *p* = 0.482 OR = 0.61 [0.17–2.14], *p* = 0.552 OR = 0.73 [0.24–2.22], *p* = 0.581 OR = 1.57 [0.56–4.39], *p* = 0.454 OR = 1.73 [0.63–4.74], *p* = 0.307
Risk factors, median (IQR)			
Systolic blood pressure Diastolic blood pressure Plasma glucose Plasma cholesterol	155 (103–207) 81 (60–120) 6.7 (17.0–4.6) 4.5 (1.9–6.5)	149.5 (110–220) 81.5 (46–115) 7.14 (1.2–22.1) 5.3 (1.7–7.1)	*p* = 0.139 *p* = 0.303 *p* = 0.904 *p* = 0.992
Anticoagulation on admission, *n* (%)	4 (7.8%)	5 (16.1%)	OR = 0.45 [0.11–1.83], *p* = 0.288
Vitamin K antagonist Underdosed VKA † Direct oral anticoagulant No acute DOAC effect ‡ LMWH	3 (75.0%) 3 (100%) 1 (25.0%) 1 (100%) 0 (0.0%)	5 (100.0%) 5 (100.0%) 0 (0.0%) 0 (0.0%) 0 (0.0%)	OR = 0.21 [0.01–6.82], *p* = 0.444 OR = 0.64 [0.01–40.1], *p* = 1 *p* = 0.444 *p* = 1 *p* = 1
pre-mRS score, median (IQR)	0 (0–5)	0 (0–4)	*p* = 0.569
pre-mRS score, *n* (%)			
0 1 2 >2	42 (82.4%) 3 (5.9%) 2 (3.9%) 4 (7.8%)	23 (74.2%) 3 (9.7%) 1 (3.2%) 4 (12.9%)	OR = 1.62 [0.55–4.78], *p* = 0.410 OR = 0.58 [0.11–3.09], *p* = 0.668 OR = 1.22 [0.11–14.1], *p* = 1 OR = 0.57 [0.13–2.48], *p* = 0.469
NIHSS score on admission, median (IQR)	10 (1–36)	9 (0–39)	*p* = 0.857

Abbreviations: BT = bridging therapy, d-MT = direct mechanical thrombectomy, OR = (common) odds ratio, IQR = interquartile range, * known or newly diagnosed, VKA = vitamin K antagonist, † defined as an INR ≤ 1.7, DOAC = direct oral anticoagulant, ‡ defined as a clotting time through whole blood viscoelastic testing (ClotPro) < 80 s (factor Xa inhibitors) and <100 s (direct IIa inhibitors), LMWH = low molecular weight heparin, pre-mRS = pre-morbidity modified Rankin Scale, NIHSS = National Institute of Health Stroke Scale.

**Table 3:** Imaging and stroke characteristics of the 31 patients in the d-MT group without contraindications for IVT and 51 patients treated with BT.

	BT (*n* = 51)	d-MT (*n* = 31)	*p*-Value/OR [95% CI]
mASPECTS, median (IQR)	9 (5–10), *n* = 46	8 (5–10), *n* = 27	*p* = 0.027
mASPECTS, *n* (%)			
5 6 7 8 9 10	1 (2.2%) 2 (4.4%) 2 (4.4%) 9 (19.6%) 12 (26.1%) 20 (43.5%)	3 (11.1%) 1 (3.7%) 5 (18.5%) 5 (18.5%) 7 (25.9%) 6 (22.2%)	OR = 0.18 [0.02–1.80], *p* = 0.140 OR = 1.18 [0.10–13.7], *p* = 1 OR = 0.20 [0.04–1.11], *p* = 0.093 OR = 1.07 [0.32–3.60], *p* = 1 OR = 1.01 [0.34–2.98], *p* = 1 OR = 2.69 [0.92–7.92], *p* = 0.081
mCTA collateral score, *n* (%)	*n* = 50	*n* = 26	
4–5 2–3 0–1	36 (72.0%) 13 (26.0%) 1 (2.0%)	17 (65.4%) 9 (34.6%) 0 (0.0%)	OR = 1.36 [0.49–3.76], *p* = 0.604 OR = 0.66 [0.24–1.85], *p* = 0.416 *p* = 1
TOAST classification, *n* (%)			
Cardioembolic stroke Large artery atherosclerosis	24 (47.1%) 13 (25.5%)	11 (35.5%) 9 (29.0%)	OR = 1.62 [0.65–4.05], *p* = 0.361 OR = 0.84 [0.31–2.27], *p* = 0.799
Occlusion site, *n* (%)			
AC stroke PC stroke	47 (92.2%) 4 (7.8%)	28 (90.3%) 3 (9.7%)	OR = 1.26 [0.26–6.04], *p* = 1 OR = 0.79 [0.17–3.81], *p* = 1
Time metrics (min), median (IQR)			
Onset-admission Door-needle-time Door-puncture-time	82 (35–185) 43 (25–195) 126 (69–290)	88 (1–303) - 112 (30–293)	*p* = 0.704 - *p* = 0.174

Abbreviations: BT = bridging therapy, d-MT = direct mechanical thrombectomy, OR = (common) odds ratio, mASPECTS = manual Alberta Stroke Program Early CT Score, IQR = interquartile range, mCTA = multiphase computed tomography angiography, TOAST = Trial of Org 10172 in Acute Stroke Treatment, AC = anterior circulation, PC = posterior circulation.

**Table 4:** Functional and procedural results of the 31 patients in the d-MT group without contraindications for IVT and 51 patients treated with BT.

	BT (*n* = 51)	d-MT (*n* = 31)	*p*-Value/OR [95% CI]
Primary endpoint			
90-day mRS score 0–2, *n* (%) 0 1 2 Mortality at 90 days, *n* (%)	21 (45.7%) *n* = 46 15 (71.4%) 2 (9.5%) 4 (19.1%) 7 (13.7%)	5 (17.2%) *n* = 29 4 (80.0%) 1 (20.0%) 0 (0.0%) 11 (35.5%)	OR = 4.03 [1.31–12.4], *p* = 0.014 OR = 3.02 [0.89–10.3], *p* = 0.102 OR = 1.27 [0.11–14.7], *p* = 1 *p* = 0.154 OR = 0.29 [0.10–0.86], *p* = 0.029
Secondary endpoint			
72 h NIHSS score, median (IQR) NIHSS score ≤ 1, *n* (%) ∆NIHSS score ≥ 4, *n* (%) Successful recanalization (mTICI ≥ 2b), *n* (%)	3 (0–37) 17 (33.3%) 23 (45.1%) 44 (86.3%)	9 (0–39) 5 (16.1%) 9 (29.0%) 29 (93.6%)	*p* = 0.095 OR = 2.60 [0.85–7.97], *p* = 0.124 OR = 2.01 [0.78–5.20], *p* = 0.169 OR = 0.43 [0.08–2.23], *p* = 0.472
2b 2c 3 First-pass complete reperfusion, *n* (%)	5 (9.8%) 13 (25.5%) 26 (51.0%) 28 (54.9%)	7 (22.6%) 7 (22.6%) 15 (48.4%) 15 (48.4%)	OR = 0.40 [0.11–1.42], *p* = 0.200 OR = 1.32 [0.45–3.84], *p* = 0.789 OR = 1.35 [0.52–3.47], *p* = 0.632 OR = 1.30 [0.53–3.18], *p* = 0.651
Safety outcome			
Thrombus migration, *n* (%) ICH, *n* (%) HI PH SAH	9 (17.7%) 2 (3.9%) 0 (0.0%) 1 (50.0%) 1 (50.0%)	3 (9.7%) 4 (12.9%) 1 (25.0%) 2 (50.0%) 1 (25.0%)	OR = 2.00 [0.50–8.04], *p* = 0.521 OR = 0.28 [0.05–1.60], *p* = 0.193 *p* = 1 OR = 1.00 [0.03–29.8], *p* = 1 OR = 2.00 [0.05–81.0], *p* = 1

Abbreviations: BT = bridging therapy, d-MT = direct mechanical thrombectomy, OR = (common) odds ratio, mRS = modified Rankin Scale, NIHSS = National Institute of Health Stroke Scale, IQR = interquartile range, mTICI = modified Thrombolysis in Cerebral Infarction, ICH = intracranial hemorrhage, HI = hemorrhagic infarction, PH = parenchymal hemorrhage, SAH = subarachnoid hemorrhage.

**Table 5:** Multivariate analysis of predictors of functional and procedural outcomes (entire cohort).

	90-Day mRS Score 0–2	90-Day Mortality	72 h NIHSS Score	NIHSS Score ≤ 1	ΔNIHSS Score ≥ 4	mTICI ≥ 2b
IVT pre-mRS score NIHSS score 72 h NIHSS score Age mASPECTS Collateral score mTICI ≥ 2b Passes Dyslipidemia Plasma glucose Plasma sodium Onset-puncture	*p* = 0.011 *p* = 0.029 *p* = 0.015 *p* < 0.001 *p* = 0.001 *p* = 0.042 *p* = 0.138 *p* = 0.441 *p* = 0.291 *p* = 0.001 *p* = 0.450 *p* = 0.557 *p* = 0.152	*p* = 0.021 *p* = 0.028 *p* = 0.003 *p* < 0.001 *p* = 0.004 *p* = 0.002 *p* = 0.010 *p* = 1 *p* = 0.549 *p* = 0.159 *p* = 0.032 *p* = 0.233 *p* = 0.580	*p* = 0.090 *p* = 0.357 *p* < 0.001 - *p* = 0.140 *p* < 0.001 *p* = 0.014 *p* = 0.080 *p* = 0.019 *p* = 0.001 *p* = 0.144 *p* = 0.047 *p* = 0.836	*p* = 0.090 *p* = 0.223 *p* = 0.060 - *p* = 0.005 *p* = 0.120 *p* = 0.013 *p* = 0.256 *p* = 0.044 *p* = 0.035 *p* = 0.647 *p* = 0.448 *p* = 0.013	*p* = 0.132 *p* = 0.936 *p* = 0.004 *p* < 0.001 *p* = 0.168 *p* = 0.187 *p* = 0.026 *p* = 0.692 *p* = 0.147 *p* = 0.239 *p* = 0.435 *p* = 0.179 *p* = 0.033	*p* = 0.299 *p* = 0.473 *p* = 0.307 - *p* = 0.150 *p* = 0.072 *p* = 0.346 - *p* < 0.001 *p* = 0.183 *p* = 0.697 *p* = 0.500 *p* = 0.223

Abbreviations: mRS = modified Rankin Scale, NIHSS = National Institute of Health Stroke Scale, mTICI = modified Thrombolysis in Cerebral Infarction, IVT = intravenous thrombolysis, pre-mRS = pre-morbidity modified Rankin Scale, mASPECTS = manual Alberta Stroke Program Early CT Score.

## Data Availability

The original contributions presented in the study are included in the article and further inquiries can be directed to the corresponding author.

## List of Abbreviations

IVT	intravenous thrombolysis
MT	mechanical thrombectomy
AIS	acute ischemic stroke
LVO	large vessel occlusion
BT	bridging therapy
d-MT	direct mechanical thrombectomy
TINL	Transzlációs Idegtudományi Nemzeti Laboratórium
mRS	modified Rankin Scale
NIHSS	National Institute of Health Stroke Scale
mTICI	modified Thrombolysis in Cerebral Infarction
rtPA	recombinant tissue plasminogen activator
RCT	randomized controlled trial
CSC	comprehensive stroke center
PSC	primary stroke center
AHS	acute hemorrhagic stroke
TIA	transient ischemic attack
SC	standard care
NCCT	non-contrast computed tomography
CTA	CT angiography
CTP	CT perfusion
ICA	internal carotid artery
ACA	anterior cerebral artery
PCA	posterior cerebral artery
mASPECTS	manual Alberta Stroke Program Early CT Score
mCTA	multiphase computed tomography angiography
TOAST	Trial of Org 10172 in Acute Stroke Treatment
DSA	digital subtraction angiography
HT	hemorrhagic transformation
ICH	intracranial hemorrhage
ECASS	European Cooperative Acute Stroke Study
HI	hemorrhagic infarction
PH	parenchymal hemorrhage
SAH	subarachnoidal hemorrhage
SPSS	Statistical Product and Service Solutions
SD	standard deviation
IQR	interquartile range
OR	odds ratio
CI	confidence interval
aICH	asymptomatic intracranial hemorrhage
sICH	symptomatic intracranial hemorrhage
CRP	C-reactive protein
